# Experimental feeding of *Sergentomyia* *minuta* on reptiles and mammals: comparison with *Phlebotomus* *papatasi*

**DOI:** 10.1186/s13071-023-05758-5

**Published:** 2023-04-13

**Authors:** Lucie Ticha, Vera Volfova, Jairo Alfonso Mendoza-Roldan, Marcos Antonio Bezerra-Santos, Carla Maia, Jovana Sadlova, Domenico Otranto, Petr Volf

**Affiliations:** 1 Department of Parasitology, Faculty of Science, Charles University, Prague, Czech Republic; 2 Department of Veterinary Medicine, University of Bari Aldo Moro, Valenzano, Italy; 3 Global Health and Tropical Medicine, Instituto de Higiene e Medicina Tropical, Universidade NOVA de Lisboa, Lisbon, Portugal; 4 Department of Pathobiology, Faculty of Veterinary Science, Bu-Ali Sina University, Hamedan, Iran

**Keywords:** Sand flies, *Sergentomyia*, *Phlebotomus*, Feeding preferences, *Leishmania*

## Abstract

**Background:**

*Sergentomyia* *minuta* (Diptera: Phlebotominae) is an abundant sand fly species in the Mediterranean basin and a proven vector of reptile parasite *Leishmania* (*Sauroleishmania*) *tarentolae*. Although it feeds preferentially on reptiles, blood meal analyses and detection of *Leishmania* (*Leishmania*) *infantum* DNA in wild-caught *S.* *minuta* suggest that occasional feeding may occur on mammals, including humans. Therefore, it is currently suspected as a potential vector of human pathogens.

**Methods:**

A recently established *S.* *minuta* colony was allowed to feed on three reptile species (i.e. lizard *Podarcis* *siculus* and geckos *Tarentola* *mauritanica* and *Hemidactylus* *turcicus*) and three mammal species (i.e. mouse, rabbit and human). Sand fly mortality and fecundity were studied in blood-fed females, and the results were compared with *Phlebotomus* *papatasi*, vector of *Leishmania* (*L*.) *major*. Blood meal volumes were measured by haemoglobinometry.

**Results:**

*Sergentomyia* *minuta* fed readily on three reptile species tested, neglected the mouse and the rabbit but took a blood meal on human. However, the percentage of females engorged on human volunteer was low in cage (3%) and feeding on human blood resulted in extended defecation times, higher post-feeding mortality and lower fecundity. The average volumes of blood ingested by females fed on human and gecko were 0.97 µl and 1.02 µl, respectively. *Phlebotomus* *papatasi* females readily fed on mouse, rabbit and human volunteer; a lower percentage of females (23%) took blood meal on the *T.* *mauritanica* gecko; reptilian blood increased mortality post-feeding but did not affect *P.* *papatasi* fecundity.

**Conclusions:**

Anthropophilic behaviour of *S.* *minuta* was experimentally demonstrated; although sand fly females prefer reptiles as hosts, they were attracted to the human volunteer and took a relatively high volume of blood. Their feeding times were longer than in sand fly species regularly feeding on mammals and their physiological parameters suggest that *S.* *minuta* is not adapted well for digestion of mammalian blood. Nevertheless, the ability to bite humans highlights the necessity of further studies on *S.* *minuta* vector competence to elucidate its potential role in circulation of *Leishmania* and phleboviruses pathogenic to humans.

**Graphical abstract:**

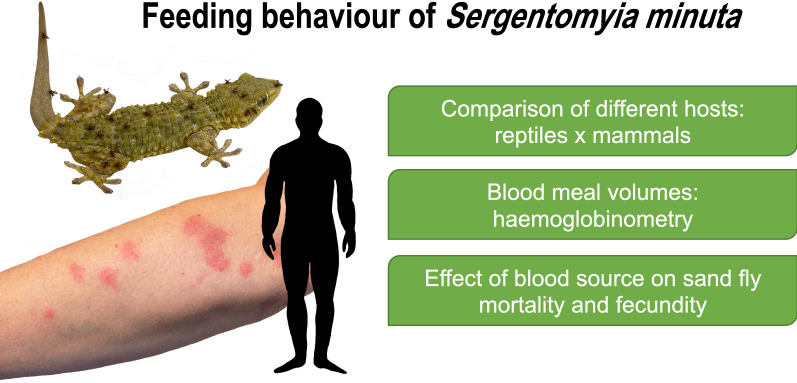

**Supplementary Information:**

The online version contains supplementary material available at 10.1186/s13071-023-05758-5.

## Background

Phlebotomine sand flies (Diptera: Psychodidae) are hematophagous insects of major medical and veterinary importance. Among more than 900 described sand fly species, approximately 100 are proven or suspected vectors of *Leishmania* protozoa, bacteria (*Bartonella* spp.) and sand fly-borne viruses [1]. In the Old World, *Phlebotomus* species act as major vectors of human diseases, while *Sergentomyia* species are mainly herpetophilic and have long been associated only with the reptilian parasites of subgenus *Sauroleishmania* [2]. Although some *Sergentomyia* species are currently suspected vectors of *Leishmania* pathogenic to humans [3], their capability to transmit human pathogens is yet to be revealed, as solid field and laboratory evidence is still lacking.

*Sergentomyia* *minuta* is one of the most abundant sand fly species in Mediterranean basin and the main vector of *Leishmania* (*Sauroleishmania*) *tarentolae*, a non-pathogenic parasite of geckos [4, 5]. Although *S*. *minuta* feeds preferentially on reptiles, blood meal analyses indicate it may occasionally feed on mammals, including humans [6–11]. Moreover, DNA of two mammalian parasites, *Leishmania* (*Leishmania*) *major* and *L.* (*L.*) *infantum* has been detected in *S.* *minuta* repeatedly [9, 12–17]. RNA of *Toscana* *phlebovirus* (TOSV), a causative agent of sporadic outbreaks of acute human encephalitis and meningoencephalitis in Mediterranean countries, was detected in wild-caught females of *S.* *minuta* in France [18]. Although molecular detection alone is not sufficient evidence to incriminate a sand fly as vector, all these findings raised questions about the spectrum of *S.* *minuta* hosts and its role in the transmission cycle of phleboviruses and *Leishmania* infecting humans and other mammals.

The aim of this study was to investigate the feeding behaviour of *S.* *minuta* in different reptilian and mammalian hosts. The effect of various blood sources on *S.* *minuta* mortality and fecundity was also studied. The results were compared with *Phlebotomus* *papatasi*, a species widespread in Europe, Africa and Asia and well known as human-biting pest [19].

## Methods

### Sand flies

The colonies of *Sergentomyia* *minuta* (originating from Portugal) and *Phlebotomus* *papatasi* (originating from Turkey) were established at the Department of Parasitology, Charles University, in 2019 and 2005, respectively. *Sergentomyia* *minuta* colony was maintained feeding on leopard geckos (*Eublepharis* *macularius*), while *P.* *papatasi* was routinely maintained on BALB/c mice. During the experiments, sand flies were kept in the insectaries of the Department of Parasitology, Charles University, and the Department of Veterinary Medicine, University of Bari. Colonies were maintained at 24–26 °C, 55–70% humidity, with 14 h light/10 h dark photoperiod, and offered 50% sucrose ad libitum, as described previously [20].

### Mammals and reptiles

Three species of mammals were tested, including a human volunteer (co-author Volfova), mice and rabbits. BALB/c mice originating from AnLab s.r.o. (Harlan Laboratories, USA) were maintained in T3 breeding containers (Velaz) equipped with bedding (German Horse Span, Pferde a.s.) and breeding material (Woodwool) and provided with a standard feed mixture (ST-1, Velaz) and water ad libitum, with a 12 h light/12 h dark photoperiod, at 22–25 °C and 40–60% humidity. NZW rabbits (originating from AnLab s.r.o.) were kept in breeding boxes (Velaz) equipped according to guidelines and legislation, provided with a standard feeding mixture for rabbits (Biopharm), hay (Krmne smesi Kvidera) and water ad libitum.

Three reptile species were offered as hosts to test sand fly feeding, with two species of geckos (Moorish gecko *Tarentola* *mauritanica*; Mediterranean house gecko *Hemidactylus* *turcicus*) and a lacertid lizard (Italian wall lizard *Podarcis* *siculus*) being compared. Animals were captured and maintained at the Department of Veterinary Medicine, University of Bari, as part of a study on *Leishmania* spp. in Mediterranean reptiles [21].

### *Sergentomyia minuta* feeding on a gecko and a human: assessment of blood meal volumes and sand fly fecundity

During the establishment of the *S.* *minuta* colony, several potential hosts for its blood-feeding were tested. Preliminary experiments revealed that, in addition to the geckos, *S.* *minuta* females also feed on human; therefore, its anthropophilic behaviour was further investigated. Routine maintenance of the *S.* *minuta* colony was done on leopard gecko (*E.* *macularius*), which is used in our faculty as a laboratory animal. However, its natural area of distribution differs from that of *S.* *minuta*; therefore, in feeding preferences experiments, we replaced it with three Mediterranean reptiles.

To compare blood meal volumes and fecundity on reptile versus mammalian blood, *S.* *minuta* females (5 days old) were fed either on a male leopard gecko (*E.* *macularius*) or on a forearm of the human volunteer. In human, a feeding chamber was used to increase numbers of *S.* *minuta* fully fed by human blood. The type of feeding chamber was described and depicted previously [22]. Briefly, 20 females were transferred into a plastic tube (diameter 3 cm) covered with fine gauze and placed onto an elbow area of a human arm for 2 h. The same relatively long exposure time (2 h) was used in gecko to allow the females to feed to repletion. Two independent trials were performed.

Haemoglobinometry was used to measure the blood meal volumes taken by individual *S.* *minuta* females. During blood-feeding, sand flies concentrate ingested proteins because of prediuresis; thus, gravimetry might lead to underestimated results. Haemoglobinometry is independent of diuresis and provides more precise estimation of the ingested blood meal volume [23, 24]. For haemoglobin assay, individual guts without Malpighian tubules were dissected 1 h post-blood meal (PBM) and transferred into microtubes with 500 μl of distilled water in batches of ten guts per sample. The samples were stored at − 70 °C until use. After thawing the samples were thoroughly homogenized and then analysed using Haemoglobin Assay Kit (MAK115, Sigma-Aldrich) following the manufacturer’s instruction. Afterwards, 50 μl of homogenate was loaded per well in triplications. The resulting haemoglobin content per gut was compared to the haemoglobin concentration measured in the host blood (same gecko and human individuals as used for experimental feeding).

The second group of females, fully fed on either gecko or human, was maintained in cages under standard conditions until defecation and dissected in buffered saline, and mature oocytes were counted under a Leica M205 FA stereomicroscope. The experiment was repeated twice.

### *Sergentomyia minuta* and *Phlebotomus papatasi* feeding on reptiles and mammals: comparison of mortality and fecundity

In experiments with reptiles, sand fly females (i.e. *n* = 50, 5–7 days old) were separated into nylon cloth cages and left there for acclimatisation for 20 min. A small number of sand fly males (< 10) was used in each group. Reptiles were placed individually into cages, and sand flies were allowed to feed in darkness, at 23–26 °C, for 2 h. In mammalian experiments the methodology was modified in the following way: BALB/c mouse anaesthetized with ketamine/xylazine (62.5 mg/kg and 25 mg/kg, respectively), mechanically immobilized NZW rabbit and forearm of the human volunteer were positioned in the cages, and sand flies were allowed to feed on the hosts for 1 h (because of the use of anaesthesia in mice).

Approximately 2 h after the hosts were removed from the cages, the blood-fed females were separated into new cages, kept under standard conditions as described above, and their post-blood meal mortality was monitored until defecation of blood meal remains (day 4 PBM in *P.* *papatasi* and day 6 PBM in *S.* *minuta*). The surviving sand fly females were then anaesthetized on ice and dissected in saline solution. The number of mature oocytes from 10 sand fly females per group was counted under the stereomicroscope, and the experiment was repeated twice.

### Statistical analysis

Differences in fecundity (oocytes numbers) of sand flies engorged on different hosts were tested by one-way ANOVA and multiple comparison of means using LSD post hoc test. Mortality and feeding were compared using Fisher’s exact or Pearson’s Chi-square test. All the statistical analyses were performed using SPSS software version 23.

## Results

### Life cycle parameters of *Sergentomyia minuta* colony

The whole development cycle of *S.* *minuta* in colony maintained on leopard geckos (*E.* *macularius*) at 26 °C was relatively fast; females laid first eggs 4–8 days post-blood meal (PBM) and first-instar larvae hatched 10–14 days PBM (Table 1). Development of four larval instars took about 2 weeks; first pupae were observed on days 23–28 PBM. Pupal period lasts for about 6 days and first adults emerged 4–5 weeks PBM. These life cycle parameters did not change during the maintenance of the colony, as they were almost the same in generations 1–4 and 23–25 (Table 1).

**Table 1:** Life cycle parameters of *Sergentomyia* *minuta* colony

		Days post-blood meal Mean (min/max)					
Year	Generation	Egg	1st instar larva	2nd instar larva	4th instar larva	Pupa	Adult
2019	1–4	6.75 (4–8)	11.50 (10–14)	16.10 (16–19)	22.00 (20–26)	26.20 (24–28)	32.40 (27–35)
2020	6–8	6.45 (4–7)	10.65 (9–12)	15.25 (13–19)	22.40 (21–26)	26.15 (23–30)	32.50 (30–37)
2022	23–25	6.30 (4–9)	10.65 (9–14)	16.30 (12–20)	22.05 (17–28)	27.60 (23–33)	33.30 (29–38)

Average intervals are given for three generations (with the range of average intervals for each generation)

### The blood meal volumes of *Sergentomyia minuta* feeding on a gecko and a human

The prolonged time of exposure (2 h), together with the application of the feeding chamber, resulted in a relatively high feeding rate (60%) on human (24 out of 40 females), which allowed the study of blood meal volumes and fecundity of females. The feeding rate on leopard gecko in nylon cloth cage was > 70%, similar to the routine feeding on this gecko species during regular colony maintenance (Volfova, personal communication).

No visible skin reaction was observed in geckos, even after repeated exposure to *S.* *minuta* bites. The blood meal volumes were measured in two samples of 10 fully engorged *S.* *minuta* females fed on gecko and two samples of 10 fully engorged females fed on human. Volumes ingested by *S.* *minuta* fed on human arm and gecko were similar and ranged around 1 µl (0.97 ± 0.03 µl/female and 1.02 ± 0.05 µl/female, respectively). As the result of such relatively big blood meals, the fully fed females were scarcely able to fly and usually only crawled out of the host.

Considerable differences were, however, found in the feeding process; on the gecko, females started to feed within 5 min and the mean feeding time to repletion was 45 min. On the other hand, feeding on human was delayed; *S.* *minuta* females started to feed in an interval from 5 to > 60 min after beginning of exposure. Unlike feeding on the gecko, the females fed on the human often interrupted feeding and needed several attempts to full engorgement.

All females fed on the gecko defecated by day 4 PBM. In contrast, defecation of females fed on the human was delayed for 2 days (i.e. by day 6 PBM). Post-feeding mortality was also higher in the females fed on the human host than in those fed on the reptile (25% versus 15%).

**Fig. 1:**
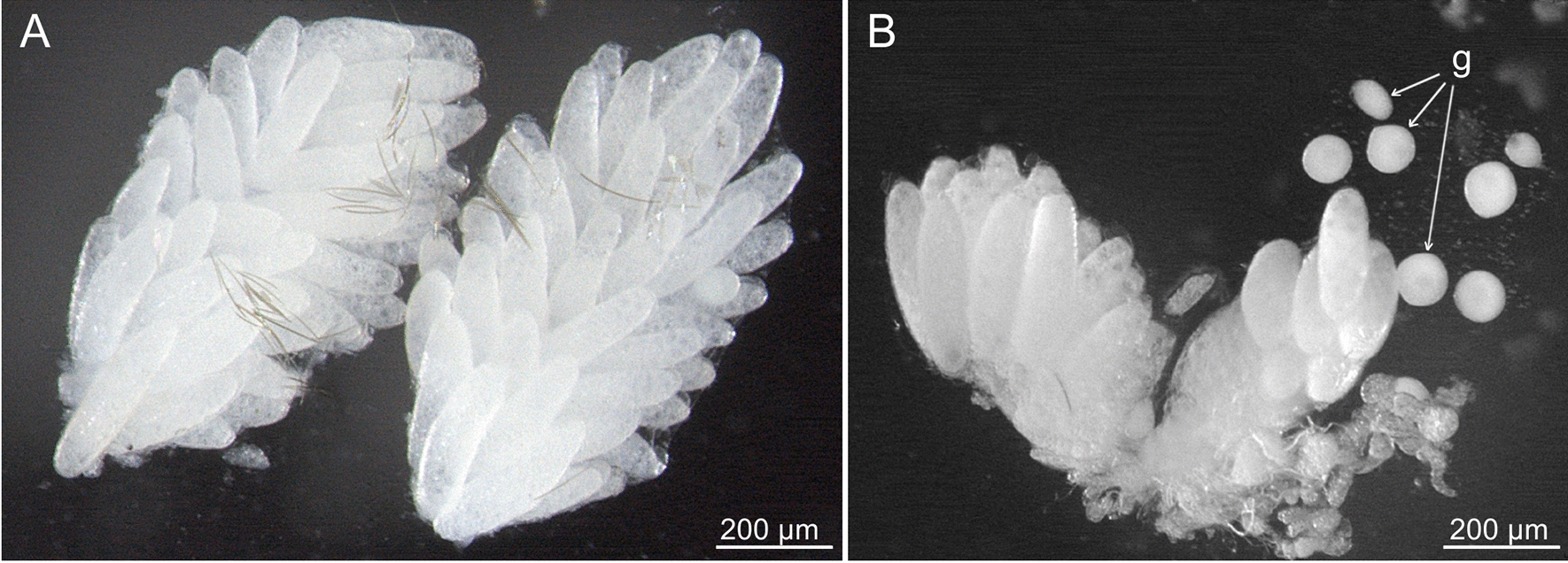
Dissected ovaria of *Sergentomyia* *minuta* females fed on gecko (**A**) and human (**B**). The number of developing oocytes was high in females fed on reptile; gregarine gamonts (g) were frequently found in the females fed on human

Following defecation, ovaria were dissected, and mature oocytes were counted. All examined females (fed on either the reptile or the human host) developed mature oocytes (Fig. 1). Nevertheless, the oocyte numbers differed substantially between the experimental groups; females fed on reptile developed significantly higher numbers (average 73, range 45–108, median 76) than those fed on human (average 26, range 17–40, median 22). Interestingly, ascogregarine infection contaminating the colony was found markedly elevated in the sand fly females fed on human blood (Fig. 1B).

### Mortality and fecundity of *Sergentomyia minuta* and *Phlebotomus papatasi* feeding on mammals

**Fig. 2:**
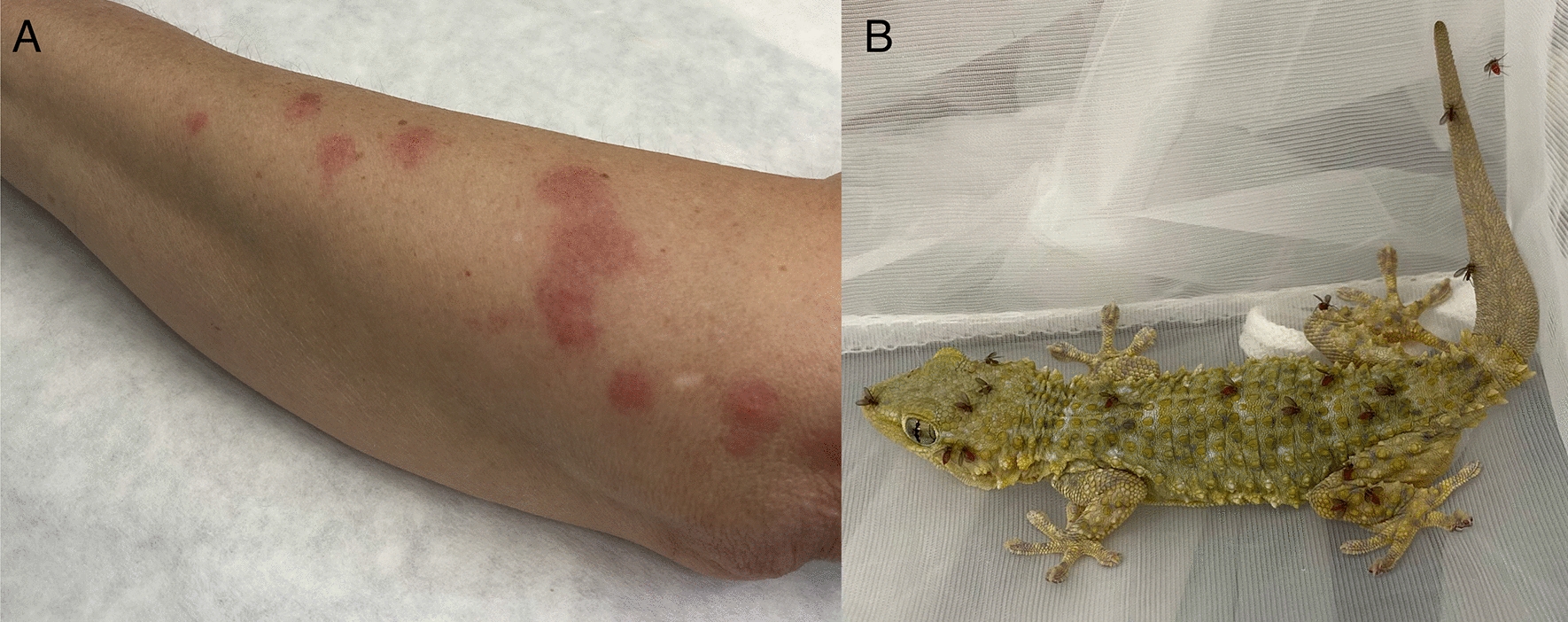
Skin hypersensitivity reaction to *Sergentomyia* *minuta* bites in human volunteer repeatedly exposed to this sand fly species (reaction 24 h post-exposure) (**A**) *S.* *minuta* females feeding on *Tarentola* *mauritanica* gecko (**B**)

**Fig. 3:**
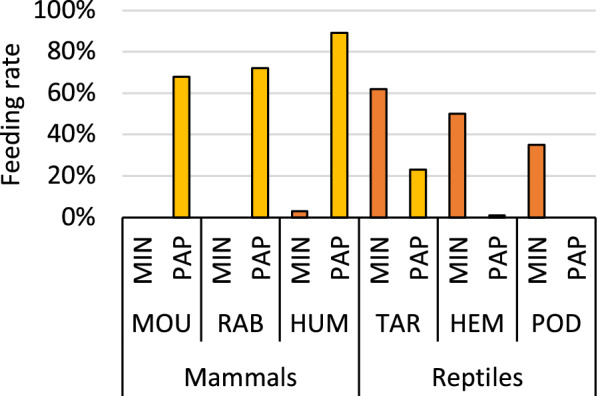
Feeding rate of *Sergentomyia* *minuta* and *Phlebotomus* *papatasi* on different mammals and reptiles. MIN *Sergentomyia* *minuta*, PAP *Phlebotomus* *papatasi*, MOU BALB/c mouse, RAB NZW rabbit, HUM human volunteer, TAR *Tarentola* *mauritanica*, HEM *Hemidactylus* *turcicus*, POD *Podarcis* *siculus*

*Sergentomyia* *minuta* females were attracted to the hand and forearm of the human volunteer placed in a nylon cloth cage several minutes after exposure. Although numerous sand fly attempts of bite were recorded (Fig. 2A), only a negligible number of females (3%) engorged on the human volunteer under these conditions (Table 2, Fig. 3). Females were feeding mostly on parts with softer skin, typically on the back of the hand, on the elbow or between the fingers. Contrarily, no *S.* *minuta* females fed on mouse or rabbit, and these hosts were completely ignored by this sand fly species (Table 2, Fig. 3). *Sergentomyia* *minuta* bites did not cause any visible effects on naive individual (human); however, repeated exposure resulted in pronounced skin hypersensitivity with maximum reaction 24–72 h post-feeding (Fig. 2A).

**Table 2:** Feeding of *Sergentomyia* *minuta* and *Phlebotomus* *papatasi* on mammals and reptiles: feeding rate and the effect on sand fly mortality and fecundity

Host species	Sand fly species	Sand fly feeding: *N* engorged/*N* (%)	Mortality post-feeding: *N* dying/*N* (%)	Number of oocytes: average (range; median)
Mouse	*S.* *minuta*	0/100 (0%)		
	*P.* *papatasi*	68/100 (68%)	2/68 (2.9%)	59.7 (26–96; 60)
Rabbit	*S.* *minuta*	0/100 (0%)		
	*P.* *papatasi*	72/100 (72%)	4/72 (5.5%)	57.8 (22–101; 53)
Human	*S.* *minuta*	3/100 (3%)	1/3 (30%)	15.5 (3–28; 15.5)
	*P.* *papatasi*	89/100 (89%)	4/89 (4.5%)	50.4 (27–85; 48)
*T.* *mauritanica*	*S.* *minuta*	62/100 (62%)	4/62 (6.4%)	64.3 (12–92; 67.5)
	*P.* *papatasi*	23/100 (23%)	3/23 (13%)	55.4 (11–83; 57)
*H.* *turcicus*	*S.* *minuta*	50/100 (50%)	3/50 (6%)	52.1 (22–73; 50.5)
	*P.* *papatasi*	1/100 (1%)	1/1 (100%)	
*P.* *siculus*	*S.* *minuta*	35/100 (35%)	5/35 (14.2%)	42.4 (27–66; 40)
	*P.* *papatasi*	0/100 (0%)

The experiment confirmed that human blood had a negative effect on the digestion, mortality and fecundity of *S.* *minuta*. Females displayed a prolonged defecation period (i.e. 5–6 days post-blood feeding), their mortality post-feeding was increased to 30%, and number of developed oocytes (15,5 in average) was significantly lower compared to blood-feeding on reptilian hosts (*P* < 0.001, *F* = 11.309; Table 2; Additional file 1).

*Phlebotomus* *papatasi* females readily fed on mouse, rabbit and human volunteer with relatively high feeding rates (68, 72 and 89%, respectively; Fig. 3). The feeding process was considerably faster compared to *S.* *minuta* females. The blood meal source did not affect the mean number of *P.* *papatasi* mature oocytes (*P* = 0.914, *F* = 0.012); 56 and 55 oocytes were produced on average after feeding on mammals (mean of all three species tested) and *T.* *mauritanica* gecko, respectively (Table 2).

### Mortality and fecundity of *Sergentomyia minuta* and *Phlebotomus papatasi* feeding on reptiles

*Sergentomyia* *minuta* readily fed on all three reptile species tested, with a slight preference of geckos over the lizard (Table 2, Fig. 2B; *P* < 0.001, Chi-square = 14.646, *df* = 2). This may be due to differences in host activity: both gecko species were relatively calm throughout the experiment, whereas the lizard was more active, and thus sand flies were more disturbed while feeding. Usually, reptiles did not show any defensive behaviour and only sporadic scratching was observed when many sand fly females were feeding at the same time. Sand fly females were attracted to reptilian hosts almost immediately after the exposure, but their feeding behaviour varied: some started to feed within a few minutes while the others after dozens of minutes or even more than 1 h. Post-blood meal mortality of *S.* *minuta* was low (6–14%) and fecundity was relatively high (42–64 oocytes in average) (Table 2).

*Phlebotomus* *papatasi* females were regularly attracted to all reptilian hosts but only a small number initiated blood-feeding, as they were more easily distracted by host activity compared to *S.* *minuta* females. Of three reptile species tested, *P.* *papatasi* females took blood only on *T.* *mauritanica* gecko (feeding rate 23%), which was the calmest reptile tested.

Although reptilian blood increased *P.* *papatasi* mortality (*P* = 0.033), it did not have a significant effect on sand fly fecundity (*P* = 0.914, *F* = 0.012); females fed on *T.* *mauritanica* gecko were able to develop oocytes in relatively high numbers (average 55.4). These numbers are fully comparable to *S.* *minuta* oocyte numbers after feeding on the same host (average 64.3) or to *P.* *papatasi* oocyte numbers after feeding on mammalian hosts (average 50.4–59.7) (Table 2).

## Discussion

In this study, we demonstrated experimentally that *S.* *minuta* had an anthropophilic behaviour, being attracted to the human volunteer. Indeed, it is generally accepted that sand flies of the genus *Phlebotomus* are mostly mammalophilic and transmit *Leishmania* pathogenic to humans, while species of the genus *Sergentomyia* are referred as herpetophilic, being proven vectors of reptilian leishmaniasis [2]. However, some members of both genera have a broader host spectrum and thus availability of the hosts is an important factor to consider. For example, an extensive study in Paloich Area in the Sudan demonstrated that several *Sergentomyia* and *Phlebotomus* sand flies feed on both mammals and/or reptiles [25]. From laboratory experiments it is known that *Sergentomyia* *schwetzi* readily feeds on geckos but can feed and thrive also on mammals for many generations [26, 27]. Such an opportunistic behaviour may have important consequences, potentially opening new epidemiological scenarios for the transmission of vectored pathogens.

Feeding behaviour of *S.* *minuta* differed from that of *P.* *papatasi* and all other sand fly species tested so far. On both types of hosts (leopard gecko and human) the relatively small-sized *S.* *minuta* females were able to ingest the biggest volume of blood meal compared to other sand flies studied to date [24, 28]. In addition, the feeding was markedly prolonged when observed in other sand fly species, including *S.* *schwetzi* [23, 29, 30], which readily feeds on various mammals [26, 27]. This behaviour may reflect the adaptation of *S.* *minuta* to feeding on reptiles. In mosquitoes, similar long feeding time up to 40 min has been observed in *Culex* *territans* mosquitoes, which also primarily feed on cold-blooded vertebrates [31]. Due to the prolonged feeding time, *S.* *minuta* females might regulate and concentrate the imbibed large volume of a blood meal in a gut via prediuresis and thus supposedly compensate significantly lower haemoglobin content in reptilian erythrocytes.

*Phlebotomus* *papatasi* is well known for its aggressive behaviour in biting humans [19] and is a proven vector of *L.* (*L*.) *major* and viruses pathogenic to humans [1]. It is considered an opportunistic species, feeding on a variety of hosts, including mammals, birds and reptiles [25, 32–34]. Recently, *P.* *papatasi* was shown to be susceptible to *L*. (*S*.) *tarentolae* under laboratory conditions [35] and the demonstration of its ability to feed on *T.* *mauritanica* geckos, further supports the hypothesis of its involvement in *Sauroleishmania* transmission as a secondary vector [35, 36].

The colony of *S.* *minuta* thrives on leopard geckos (*E.* *macularius*); at standard temperature 26 °C the development of all life cycle stages was relatively fast. Comparison to other colonies maintained at the Department of Parasitology, Charles University, showed that *S.* *minuta* has the shortest generation time (i.e. 7–8 weeks). Accordingly, the larval period took approximately 2 weeks, which is about 1 week shorter than in other sand flies maintained in the same conditions [20]. In contrast to humans, no skin reactions were observed in geckos after repeated *S.* *minuta* bites.

Herpetophillic behaviour of *S.* *minuta* demonstrated in the experiments overlaps results of previous reports from the field [10, 11, 17]. Among three reptile species tested, *S.* *minuta* readily fed on geckos but was also able to feed on *P.* *siculus* lizards, from which *L*. (*S*.) *tarentolae* DNA was recently isolated [16]. This *Sauroleishmania* species has so far been described in three species of geckos, namely *Tarentola* *mauritanica*, *T.* *annularis* and *Mediodactylus* *kotschyi* [5]. The ability of *S.* *minuta* to feed on *P.* *siculus* highlights the possibility that this common lizard is involved in circulation of *L.* (*S*.) *tarentolae* in Italy.

Even more interesting is, however, the experimental confirmation that *S.* *minuta* occasionally bites mammals, particularly humans. Although *S.* *minuta* prefers reptiles as a blood meal source, females were attracted to the forearm of the human volunteer placed in the nylon cloth cage and took a blood meal. These findings correspond to the results of field surveys showing this sand fly species occasionally feeds on human blood [6–11]. In *S.* *minuta*, humans were the most frequently detected hosts apart from reptiles in different catching sites [6, 8–11]. However, it was also reported that this species feeds to a lesser extent on a relatively wide range of mammalian hosts, including large ungulates, dogs and rabbits [9]. Our experiments showed that *S.* *minuta* took a blood meal on human volunteer but completely refused to feed on a rabbit or a mouse.

If allowed to feed ad libitum, *S.* *minuta* females were able to acquire almost the same volume of blood meal on the human as on the reptile host (approximately 1 µl). Nevertheless, the digestion of human blood was prolonged, post-feeding mortality was increased, while fecundity was decreased. Similar changes of physiological parameters were observed during an unsuccessful attempt to keep a *S.* *minuta* colony by feeding on humans: females had high mortality, low fecundity and the colony died out after two generations (Volfova and Volf, unpublished). All these results suggest that S. *minuta* is not adapted to feeding on mammals and cannot digest human blood properly. Consequently, feeding on humans is more likely an opportunistic behaviour of this sand fly species, which is in striking contrast to *S.* *schwetzi* where a lineage feeding exclusively on mice was successfully established [27], and females readily feed on humans (Volfova and Volf, unpublished).

Potential involvement of *Sergentomyia* as vectors of human pathogenic *Leishmania* spp. was mentioned repeatedly [3] but reliable evidence is still lacking and all *Leishmania* parasites isolated from *S.* *minuta* so far were typed as *L*. (*S*.) *tarentolae* [5]. Interestingly, this reptilian parasite was recently also detected in humans [11, 37] and dogs [16], and thus its pathogenic potential for mammals is currently unclear [5]. Demonstration of *S.* *minuta* feeding on humans may therefore explain how *L*. (*S*.) *tarentolae* was transmitted from geckos to humans and dogs.

Laboratory experiments are crucial for vector identification. Even though promastigotes were found and *L*. (*L*.) *infantum* DNA was detected in *S.* *schwetzi* [38], it was proved experimentally that this sand fly is refractory to mammal-infecting *Leishmania* spp. [39]. Early phase of *Leishmania* infection in sand flies is a non-specific process accompanied by rapid multiplication of promastigotes in the ingested blood meal; then, defecation of blood meal remnants represents the crucial barrier in unnatural parasite-vector pairs [40]. *Leishmania* (*L*.) *infantum* promastigotes were able to develop early-stage infections even in biting midges *Culicoides* *nubeculosus*, but they were, similarly to *S.* *schwetzi*, lost during defecation, although *Leishmania* DNA was detectable up to 7 days post-infection [41].

Unfortunately, the experiments with *S.* *minuta* are limited by the fact that females refused to feed through membranes. All attempts to perform experimental infections with this species failed, although various feeding conditions were tested repeatedly; these include the use of different blood sources (i.e. sheep, rabbit and chicken blood), membranes (i.e. chick skin, gecko skin, a membrane from pig intestine) and changes of temperature and humidity in the experimental box. Therefore, the field work accompanied by direct observation on natural promastigote infection (together with parasite isolation and its typing) remains the best way to prove the involvement of *S.* *minuta* in circulation of *L*. (*L*.) *infantum* and other species pathogenic to mammals.

## Conclusions

Experimental data on the feeding behaviour of *S.* *minuta* were herein assessed for the first time. We demonstrated that *S.* *minuta* females readily took a blood meal on geckos and lizards and that the feeding times of *S.* *minuta* were significantly longer than those typical for sand fly species regularly feeding on mammals. Interestingly, despite the relatively small size of this sand fly species, the volume of ingested blood was higher than in other sand fly species tested so far. *Sergentomyia* *minuta* females refused to feed on mice and rabbits but were able to bite a human volunteer, causing pronounced skin hypersensitivity reaction in the volunteer repeatedly exposed. Digestion of human blood was prolonged, post-feeding mortality was high, and fecundity was reduced. All these findings suggest that *S.* *minuta* is not well adapted to feeding on humans and digesting human blood. However, the ability of *S.* *minuta* to bite humans raises questions about its potential role in circulation of various *Leishmania* parasites and phleboviruses.

## Supplementary Information

**Additional file 1: Table S1**. Number of developed oocytes of *Sergentomyia* *minuta* females feeding on different hosts. TAR, *Tarentola* *mauritanica*; HEM, *Hemidactylus* *turcicus*; POD, *Podarcis* *siculus*; HUM, human volunteer. **Table S2**. Comparison of *Sergentomyia* *minuta* fecundity after feeding on different hosts. TAR, *Tarentola* *mauritanica*; HEM, *Hemidactylus* *turcicus*; POD, *Podarcis* *siculus*; HUM, human volunteer.

## Data Availability

All the data are included within the article and its additional files.

## Abbreviations

PBM: Post-blood meal
TOSV: *Toscana* *phlebovirus*

## References

[1] Maroli M, Feliciangeli MD, Bichaud L, Charrel RN, Gradoni L (2013). Phlebotomine sandflies and the spreading of leishmaniases and other diseases of public health concern. Med Vet Entomol.

[2] Killick-Kendrick R, Lainson R, Rioux JA, Saf’janova VM. The taxonomy of *Leishmania*-like parasites of reptiles. In: Rioux JA, editor. *Leishmania*: Taxonomie et phylogenèse. Application Éco-epidemiologiques (Colloque International du CNRS/INSERM, 1984), MEE, Montpellier; 1986. p. 143–8.

[3] Maia C, Depaquit J. Can *Sergentomyia* (Diptera, Psychodidae) play a role in the transmission of mammal-infecting *Leishmania*?. Parasit Vectors. 2016;23:55. 10.1051/parasite/2016062. 10.1051/parasite/2016062 PMC5159824 27921993

[4] Klatt S, Simpson L, Maslov DA, Konthur Z (2019). *Leishmania*
*tarentolae*: Taxonomic classification and its application as a promising biotechnological expression host. PLoS Negl Trop Dis.

[5] Mendoza-Roldan JA, Votypka J, Bandi C, Epis S, Modry D, Ticha L (2022). *Leishmania*
*tarentolae*: a new frontier in the epidemiology and control of the leishmaniases. Transbound Emerg Dis.

[6] Maia C, Parreira R, Cristóvão JM, Freitas FB, Afonso MO, Campino L (2015). Molecular detection of *Leishmania* DNA and identification of blood meals in wild caught phlebotomine sand flies (Diptera: Psychodidae) from southern Portugal. Parasit Vectors.

[7] Bravo-Barriga D, Parreira R, Maia C, Afonso MO, Frontera E, Campino L (2016). First molecular detection of *Leishmania*
*tarentolae*-like DNA in *Sergentomyia*
*minuta* in Spain. Parasitol Res.

[8] Bennai K, Tahir D, Lafri I, Bendjaballah-Laliam A, Bitam I, Parola P (2018). Molecular detection of *Leishmania*
*infantum* DNA and host blood meal identification in *Phlebotomus* in a hypoendemic focus of human leishmaniasis in northern Algeria. PLoS Negl Trop Dis.

[9] Abbate JM, Maia C, Pereira A, Arfuso F, Gaglio G, Rizzo M (2020). Identification of trypanosomatids and blood feeding preferences of phlebotomine sand fly species common in Sicily, Southern Italy. PLoS ONE.

[10] González E, Molina R, Aldea I, Iriso A, Tello A, Jiménez M (2020). *Leishmania* sp. detection and blood-feeding behaviour of S*ergentomyia*
*minuta* collected in the human leishmaniasis focus of southwestern Madrid, Spain (2012–2017). Transbound Emerg Dis.

[11] Pombi M, Giacomi A, Barlozzari G, Mendoza-Roldan JA, Macrì G, Otranto D (2020). Molecular detection of *Leishmania* (*Sauroleishmania*) *tarentolae* in human blood and *Leishmania* (*Leishmania*) *infantum* in *Sergentomyia*
*minuta*: unexpected host-parasite contacts. Med Vet Entomol.

[12] Campino L, Cortes S, Dionísio L, Neto L, Afonso MO, Maia C (2013). The first detection of *Leishmania*
*major* in naturally infected *Sergentomyia*
*minuta* in Portugal. Mem Inst Oswaldo Cruz.

[13] Jaouadi K, Ghawar W, Salem S, Gharbi M, Bettaieb J, Yazidi R (2015). First report of naturally infected *Sergentomyia*
*minuta* with *Leishmania*
*major* in Tunisia. Parasit Vectors.

[14] Pereira S, Pita-Pereira D, Araujo-Pereira T, Britto C, Costa-Rego T, Ferrolho J (2017). First molecular detection of *Leishmania*
*infantum* in *Sergentomyia*
*minuta* (Diptera, Psychodidae) in Alentejo, southern Portugal. Acta Trop.

[15] Latrofa MS, Iatta R, Dantas-Torres F, Annoscia G, Gabrielli S, Pombi M (2018). Detection of *Leishmania*
*infantum* DNA in phlebotomine sand flies from an area where canine leishmaniosis is endemic in southern Italy. Vet Parasitol.

[16] Mendoza-Roldan JA, Latrofa MS, Iatta R, Manoj RRS, Panarese R, Annoscia G (2021). Detection of *Leishmania*
*tarentolae* in lizards, sand flies and dogs in southern Italy, where *Leishmania*
*infantum* is endemic: hindrances and opportunities. Parasit Vectors.

[17] Mendoza-Roldan JA, Zatelli A, Latrofa MS, Iatta R, Bezerra-Santos MA, Annoscia G (2022). *Leishmania* (*Sauroleishmania*) *tarentolae* isolation and sympatric occurrence with *Leishmania* (*Leishmania*) *infantum* in geckoes, dogs and sand flies. PLoS Negl Trop Dis.

[18] Charrel RN, Izri A, Temmam S, De Lamballerie X, Parola P (2006). *Toscana* virus RNA in *Sergentomyia*
*minuta* flies. Emerg Infect Dis.

[19] Lewis DJ, Ward RD, Peters W, Killick-Kendrick R (1987). Transmission and vectors. The leishmaniases in biology and medicine.

[20] Volf P, Volfova V (2011). Establishment and maintenance of sand fly colonies. J Vector Ecol.

[21] Mendoza-Roldan JA, Latrofa MS, Tarallo VD, Manoj RRS, Bezerra-Santos MA, Annoscia G (2022). *Leishmania* spp. in Squamata reptiles from the Mediterranean basin. Transbound Emerg Dis.

[22] Sadlova J, Vojtkova B, Hrncirova K, Lestinova T, Spitzova T, Becvar T (2019). Host competence of African rodents *Arvicanthis*
*neumanni*, *A.*
*niloticus* and *Mastomys*
*natalensis* for *Leishmania*
*major*. Int J Parasitol Parasites Wildl..

[23] Sadlova J, Reishig J, Volf P (1998). Prediuresis in female *Phlebotomus* sandflies (Diptera: Psychodidae). Eur J Entomol.

[24] Pruzinova K, Sadlova J, Seblova V, Homola M, Votypka J, Volf P (2015). Comparison of bloodmeal digestion and the peritrophic matrix in four sand fly species differing in susceptibility to *Leishmania*
*donovani*. PLoS ONE.

[25] Quate LW (1964). Phlebotomus sandflies of the Paloich area in the Sudan (Diptera, Psychodidae). J Med Entomol.

[26] Lawyer PG, Ngumbi PM, Anjili CO, Odongo SO, Mebrathu YM, Githure JI (1990). Development of *Leishmania*
*major* in *Phlebotomus*
*duboscqi* and *Sergentomyia*
*schwetzi* (Diptera: Psychodidae). Am J Trop Med Hyg.

[27] Polanska N, Ishemgulova A, Volfova V, Flegontov P, Votypka J, Yurchenko V (2020). *Sergentomyia*
*schwetzi*: Salivary gland transcriptome, proteome and enzymatic activities in two lineages adapted to different blood sources. PLoS ONE.

[28] Daba S, Daba A, Shehata MG, El Sawaf BM (2004). A simple micro-assay method for estimating blood meal size of the sand fly, *Phlebotomus*
*Langeroni* (Diptera: Psychodidae). J Egypt Soc Parasitol.

[29] Sant’anna MR, Nascimento A, Alexander B, Dilger E, Cavalcante RR, Diaz-Albiter HM (2010). Chicken blood provides a suitable meal for the sand fly *Lutzomyia*
*longipalpis* and does not inhibit *Leishmania* development in the gut. Parasit Vectors.

[30] Roby NH, Hussein MA, Doha SA, Ghani SA (2015). Effect of different blood sources on the feeding time of sand fly, *Phlebotomus*
*papatasi*. J Egypt Soc Parasitol.

[31] Reinhold JM, Shaw R, Lahondère C. Beat the heat: *Culex**quinquefasciatus* regulates its body temperature during blood-feeding. J Therm Biol. 2021;96:102826. 10.1016/j.jtherbio.2020.102826. 10.1016/j.jtherbio.2020.102826 33627266

[32] Adler S, Theodor O (1929). Observations on *Leishmania*
*ceramodactyli* N.SP. Trans R Soc Trop Med Hyg.

[33] Svobodova M, Sadlova J, Chang KP, Volf P (2003). Distribution and feeding preference of the sand flies *Phlebotomus*
*sergenti* and *P.*
*papatasi* in a cutaneous leishmaniasis focus in Sanliurfa, Turkey. Am J Trop Med Hyg.

[34] Palit A, Bhattacharya SK, Kundu SN (2005). Host preference of *Phlebotomus*
*argentipes* and *Phlebotomus*
*papatasi* in different biotopes of West Bengal, India. Int J Environ Health Res.

[35] Ticha L, Kykalova B, Sadlova J, Gramiccia M, Gradoni L, Volf P (2021). Development of various *Leishmania* (*Sauroleishmania*) *tarentolae* strains in three *Phlebotomus* species. Microorganisms.

[36] Ticha L, Sadlova J, Bates P, Volf P (2022). Experimental infections of sand flies and geckos with *Leishmania* (*Sauroleishmania*) *adleri* and *Leishmania* (*S*.) *hoogstraali*. Parasit Vectors.

[37] Iatta R, Mendoza-Roldan JA, Latrofa MS, Cascio A, Brianti E, Pombi M (2021). *Leishmania*
*tarentolae* and *Leishmania*
*infantum* in humans, dogs and cats in the Pelagie archipelago, southern Italy. PLoS Negl Trop Dis.

[38] Senghor MW, Niang AA, Depaquit J, Ferté H, Faye MN, Elguero E (2016). Transmission of *Leishmania*
*infantum* in the canine leishmaniasis focus of Mont-Rolland, Senegal: ecological, parasitological and molecular evidence for a possible role of *Sergentomyia* sand flies. PLoS Negl Trop Dis.

[39] Sadlova J, Dvorak V, Seblova V, Warburg A, Votypka J, Volf P (2013). *Sergentomyia*
*schwetzi* is not a competent vector for *Leishmania*
*donovani* and other *Leishmania* species pathogenic to humans. Parasit Vectors.

[40] Dostalova A, Volf P (2012). *Leishmania* development in sand flies: parasite–vector interactions overview. Parasit Vectors.

[41] Seblova V, Sadlova J, Carpenter S, Volf P (2014). Development of *Leishmania* parasites in *Culicoides*
*nubeculosus* (Diptera: Ceratopogonidae) and implications for screening vector competence. J Med Entomol.

