# Supplementary material for: Experimental feeding of Sergentomyia minuta on reptiles and mammals: comparison with Phlebotomus papatasi
Source: Parasit Vectors. 2023 Apr 13;16:126. doi: 10.1186/s13071-023-05758-5 (PMC10103492; doi:10.1186/s13071-023-05758-5)
Supplement: Supplementary file 1 — Additional file 1: Table S1. Number of developed oocytes of Sergentomyia minuta females feeding on different hosts. TAR, Tarentola mauritanica; HEM, Hemidactylus turcicus; POD, Podarcis siculus; HUM, human volunteer. Table S2. Comparison of Sergentomyia minuta fecundity after feeding on different hosts. TAR, Tarentola mauritanica; HEM, Hemidactylus turcicus; POD, Podarcis siculus; HUM, human volunteer. [file 13071_2023_5758_MOESM1_ESM.pdf]

Additional file 1

	N	Mean	Std. Deviation	Std. Error	Lower Bound	Upper Bound
TAR	20	64.30	18.264	4.084	55.75	72.85
HEM	20	52.10	13.814	3.089	45.63	58.57
POD	20	42.35	11.758	2.629	36.85	47.85
HUM	2	15.50	17.678	12.500	-143.33	174.33
Total	62	51.71	18.310	2.325	47.06	56.36

Table S1. Number of developed oocytes of *Sergentomyia minuta* females feeding on different hosts. TAR, *Tarentola mauritanica*; HEM, *Hemidactylus turcicus*; POD, *Podarcis siculus*; HUM, human volunteer.

(I) Host	(J) Host	Mean Difference (I-J)	Std. Error	Sig.	Lower Bound	Upper Bound
TAR	HEM	12.200*	4.717	.012	2.76	21.64
	POD	21.950*	4.717	.000	12.51	31.39
	HUM	48.800*	11.061	.000	26.66	70.94
HEM	TAR	-12.200*	4.717	.012	-21.64	-2.76
	POD	9.750*	4.717	.043	.31	19.19
	HUM	36.600*	11.061	.002	14.46	58.74
POD	TAR	-21.950*	4.717	.000	-31.39	-12.51
	HEM	-9.750*	4.717	.043	-19.19	-.31
	HUM	26.850*	11.061	.018	4.71	48.99
HUM	TAR	-48.800*	11.061	.000	-70.94	-26.66
	HEM	-36.600*	11.061	.002	-58.74	-14.46
	POD	-26.850*	11.061	.018	-48.99	-4.71

Table S2. Comparison of *Sergentomyia minuta* fecundity after feeding on different hosts. TAR, *Tarentola mauritanica*; HEM, *Hemidactylus turcicus*; POD, *Podarcis siculus*; HUM, human volunteer.
